# Can AI developers avoid bias in public health applications?

**DOI:** 10.3389/fpubh.2025.1752729

**Published:** 2026-01-13

**Authors:** Rebekah J. Harms, Rachel A. Ankeny, Lucy Carter, Aditi Mankad, Jackie Leach Scully

**Affiliations:** 1 Disability Innovation Institute, UNSW Sydney, Sydney, NSW, Australia; 2 Advanced Engineering Biology Future Science Platform, CSIRO, Brisbane, QLD, Australia; 3 Philosophy Group, Wageningen University, Wageningen, Netherlands; 4 CSIRO Environment, CSIRO, Brisbane, QLD, Australia

**Keywords:** artificial intelligence, bias, engineering biology, public health, responsibility

## Abstract

Developments in the field of engineering biology and artificial intelligence have made it increasingly possible to deliver personalised treatments which are tailored to the individual and can help prevent illnesses before they occur. While such advancements have important implications for public health, the use of AI-enabled personalised treatments comes with potential downsides, not least of which is the potential for bias which may cause harm to certain subpopulations. As one of the key actors in the AI development pipeline, developers are ideally placed to ensure that treatments are designed in an equitable manner. However, existing bias mitigation strategies often fail to consider the practical challenges faced by developers which can significantly impact their abilities to detect and remove bias from any treatments which they help to design. In this paper, we highlight some of the practical challenges that developers face in mitigating bias. We also consider the implications of acknowledging such limitations for attributing responsibility related to bias mitigation.

## Introduction

Artificial intelligence (AI) is poised to fundamentally alter the ways in which we approach public health, particularly given the rise of AI-enabled personalised medicine which can not only facilitate the delivery of tailored treatments to wider segments of the community but can also make it easier to prevent illnesses from occurring in the first place. The use of AI-enabled biosensors, for example, can lead to the earlier identification of illnesses and motivate individuals to behave in ways that may improve their health (1, 2). Personalised medical applications can extend treatment to a wider range of subpopulations, which can in turn contribute to the lessening of health inequalities (3). One example is engineered biosensors that can detect and respond to specific targets under relatively low resource conditions and require simple infrastructure, enabling practitioners to monitor the health conditions of those in remote areas who may have limited access to healthcare (1). AI offers enormous value to the delivery of personalised treatments due to its abilities to analyse large quantities of genomic data, make risk predictions, and conduct real-time monitoring (3, 4).

To deliver personalised medicine, AI technologies must be underpinned by scientific knowledge stemming from fields including engineering biology, which involves the application of engineering principles to the development of biological products and services (5, 6). Data-driven molecular design, a key area of research within engineering biology, uses AI to rapidly design and predict the functions of biological molecules, which has important therapeutic applications (7). Techniques used within engineering biology, such as next-generation sequencing, cell and gene therapy, pharmacogenomic testing, and microencapsulation, are also central to advancements in personalised medicine (8). When tailored with AI, discoveries in the field of engineering biology have the potential to usher in radically new ways of delivering healthcare that are more effective, targeted, and efficient.

While AI-enabled personalised medicine can add significant value to the health system—offering benefits such as faster diagnoses, more effective drug treatments, and improved patient outcomes—it is important that those who develop and administer such treatments do so responsibly to create the highest levels of benefit for the greatest number of people, and with the least risk of harm. AI bias is a particularly challenging obstacle that can occur at all stages of the development pipeline, from algorithm design to algorithm validation and clinical implementation (9). Algorithms may perform unequally in different subpopulations if not trained on datasets which are representative of diverse demographic and genetic factors (9). In addition, AI designers may feed their own biases into algorithms which can shape their outputs, such as when different developers inconsistently assign meaning to the data on which an algorithm is trained (10).

Although AI developers are only one type of actor in the much broader healthcare landscape, it is useful to consider what actions they can take to mitigate bias in the design of AI-enabled treatments (11). Developers are generally considered to be those who design and build AI algorithms, and have input throughout the development pipeline, including data preparation, algorithm design, and both pre- and post-deployment model evaluation (12). Developers typically are data scientists, AI scientists, or AI engineers (12). As one of the key actors along the development pipeline, developers have an important role to play in mitigating bias and ensuring that algorithms are designed in an equitable manner. Yet developers are often constrained by practical challenges which limit their capacity to engage in bias mitigation activities. In this paper, we critically explore the practicality of common bias mitigation strategies by highlighting some of the challenges which developers face in designing non-biased algorithms.

## Current bias mitigation strategies

**Figure 1:**
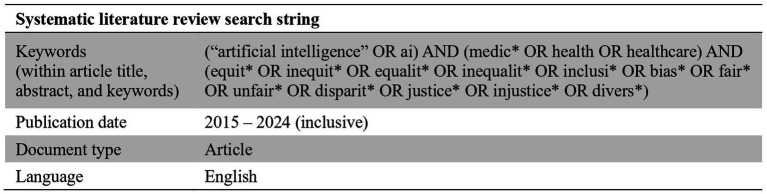
Search parameters for systematic literature review.

**Figure 2:**
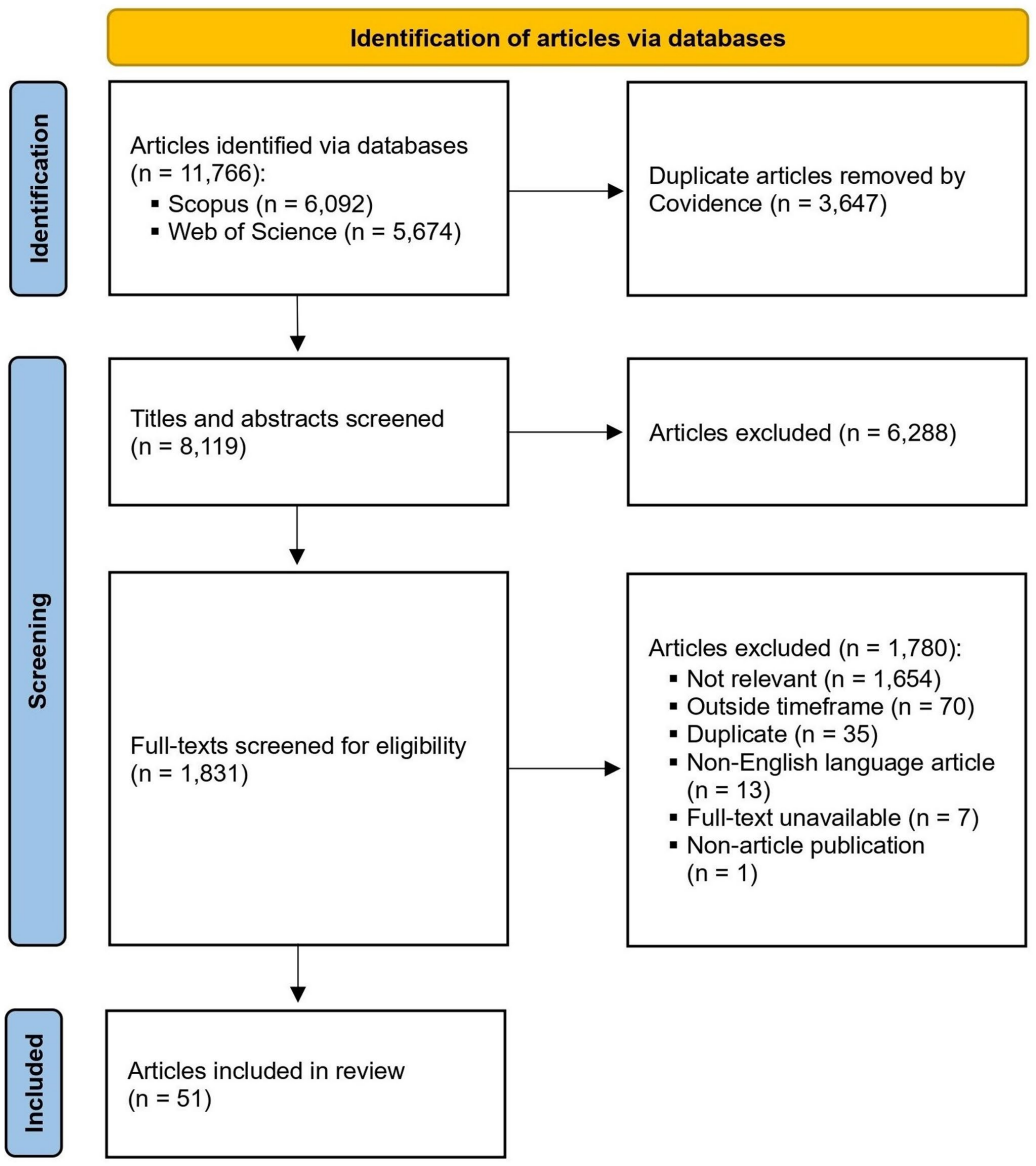
PRISMA 2020 flow diagram (49).

Before delving into the practical challenges faced by developers, it is useful to consider the current research landscape on developer-oriented bias mitigation strategies. We conducted a systematic literature review over the past 10 years (2015–2024) exploring bias mitigation strategies proposed for implementation by AI developers (13). By “bias mitigation strategies,” we mean any actions that can be taken by developers to reduce the likelihood or severity of bias in the design of an AI algorithm. The search was performed specifically within the literature in the field of healthcare, and captured any articles discussing concepts related to bias, such as fairness, equity, inclusivity, and justice. Articles were included if they provided solutions for bias which were directly targeted at AI developers (see Figures 1, 2 for an overview of the review process).

Analysis of the 51 articles included in the review showed that bias mitigation strategies tend to be grouped around seven key themes. Many articles argue that development teams should be composed of a diverse range of individuals, including from different demographic backgrounds and with diverse areas of expertise (14, 15). Some claim that developers need more training and education on bias mitigation (16, 17). Responsibility is placed on developers to be aware of potential sources of bias and to be reflexive about their own biases (18, 19). Many highlight the importance of training algorithms on datasets that represent diverse and underserved subpopulations (20, 21). Several claim that collaborating with end users and beneficiaries could help developers identify a wider range of potential biases (22, 23). Monitoring is another key theme, with emphasis on the need for developers to regularly evaluate algorithm performance during the design and implementation stages (20, 24). Finally, transparency around algorithm performance is seen as an important part of the bias mitigation process (25, 26).

All of these strategies have value in a public health context. For example, by ensuring that the data which are used to train algorithms are representative of diverse populations, developers can help to improve community-wide health outcomes (11). Being transparent about the strengths and limitations of a given algorithm, including any potential sources of bias, is also critical, particularly given the increasingly hands-on approach that many people take towards their own healthcare (11, 27).

The bias mitigation strategies identified in the literature are useful insofar as they provide general guidelines for developers to follow when seeking to minimise bias. However, the reviewed articles generally fail to suggest how these strategies can be operationalised in real-world settings. Almost all of the reviewed articles tackle the issue of AI bias from a theoretical standpoint, with only 18% of the articles basing their arguments in empirical findings. In addition, the majority of articles (63%) address the issue of bias in healthcare as a whole, rather than within a particular field of medicine, with only one of the reviewed articles specifically addressing ways that developers can mitigate bias when using AI for public health-related purposes, namely Flores, Kim, and Young who discuss some of the actions that developers can take to minimise bias when designing algorithms for public health surveillance purposes (10). What is largely left out of their discussion, however, is a consideration of the constraints that developers face which may prevent them from fully adopting the outlined strategies. Without understanding the contexts within which developers work, it is difficult to appreciate the practical limitations that developers face in implementing these strategies in real-world settings. In the following section, we provide some examples illustrative of the challenges that developers face in undertaking bias mitigation.

## Limitations of the AI development environment

Despite widespread calls for developers to access diverse data, research has shown that health datasets often lack diversity in genetic and demographic data (28, 29). This gap is problematic, as an individual’s genetic makeup, and demographic factors such as sex and age, have direct impacts on health outcomes, meaning that algorithms trained on under-representative data may not be as effective for certain subpopulations (30, 31). While developers can use techniques such as oversampling or ensemble learning to minimise bias from under-representative datasets, significant ambiguity still exists around how to deal with missing data (32). Should developers hold off on developing algorithms until they have access to better data, or should they continue to develop treatments that are known to deliver health benefits for only some segments of the community (33)? Similar questions can be asked about the merits of using synthetic data to fill gaps in existing datasets, particularly given concerns that this approach can potentially reinforce biases and undermine the consent process (34).

Developers are also constrained by decisions made upstream in the collection and storage of health data. Placing the onus on developers to access diverse data is problematic as it assumes that they *can actually* do so. On the other hand, those who create and manage datasets tend to be better situated than developers to shape the types of health data that are collected (35). For example, to protect data subjects’ privacy and for logistical reasons, developers may need to access data through federated learning systems which enable them to test their algorithms on a dataset without directly accessing the data itself (36, 37). The managers of the datasets, rather than the developers themselves, are thus responsible for deciding what types of data are made available. Upstream decisions around data collection therefore have significant impacts on whether a developer is able to design a representative algorithm. Questions remain about how to strike the appropriate balance between ensuring that datasets are diverse and protecting the privacy of data subjects, particularly when dealing with sensitive genetic data. Should datasets be scrubbed entirely of personal information such as ethnicity and socioeconomic status to protect patients’ privacy, or should such information be retained in order to better judge the fairness of a particular algorithm (38, 39)? Again, dataset managers are best placed to answer such questions given their responsibility over data dissemination.

Collaborating with the beneficiaries of AI-enabled treatments is another key bias mitigation strategy mentioned in the literature that comes with its own challenges. It is vital that developers produce algorithms which reflect the values and needs of the community (40). This type of outcome can be achieved by engaging with the beneficiaries of AI-enabled treatments in participatory ways, such as processes of co-design (41). Taking a diverse range of views into account can help developers to minimise their personal biases and identify sources of bias that may have been overlooked (41). Engaging the community can also help combat a common critique levelled against public health initiatives, namely that they risk devaluing individual choice in healthcare (42).

Despite the benefits of community engagement, developers may not have the time, skills, or resources to systematically collaborate with members of the public each time a new treatment is being developed (43). Other actors in the AI development pipeline may be better placed to engage with relevant publics. Healthcare practitioners, for example, have direct access to patients, making them ideally placed to provide insights on the healthcare needs and values of the wider public (44). Ethicists and social scientists can also provide insights into community values, particularly based on their empirical research, including how people want their data to be used and stored (45). Developers may thus have to rely on the insights of intermediaries who have better access to the public, particularly when they do not have the resources or capacity to undertake direct engagement themselves.

Implicit within much of the literature on developer-oriented bias mitigation strategies is the assumption that developers maintain oversight and control over their algorithms throughout all stages of the development process (44). Yet in some cases, it is unreasonable to expect individual developers to be responsible for biases that become apparent once an algorithm has been clinically deployed. It is well recognised that bias which was not present during the design stage can emerge during model deployment (9). End users, for example, may overrely on a model’s findings, leading to automation bias (46). These biases may even feed back into the algorithm if it has been programmed to learn from end users’ interpretations (46). One way of mitigating these biases is to ensure that developers maintain oversight of their models during the implementation stage to ensure that they are working as desired and any new or existing biases are identified and removed (44). Yet in practise, developers may only be engaged in upstream model design and may not be involved in the commercialisation of a given AI-enabled treatment, making it impossible for them to rectify such biases.

Finally, developers may be hampered by time and resource constraints that prevent them from fully understanding how an algorithm has been designed. Designing a bespoke algorithm from scratch is an expensive and time-consuming process and may not be necessary if an existing model that can be adapted is readily available (47). In such circumstances, developers may not be fully aware how an algorithm has been designed, making it difficult to detect whether any biases have been built into the model (48). Expecting developers to be aware of such biases can be problematic, particularly when algorithm owners are not forthcoming about how a given algorithm has been designed and on which subpopulations it has been tested. Greater clarity about the appropriate attribution of responsibility in such cases is urgently needed. Should developers who take advantage of existing algorithms be responsible for earlier design choices which lead to bias, or should algorithm owners be held accountable for the downstream implications of their model? Most importantly, how can bias be identified and mitigated in these typical types of development processes?

## Gaps left to address

With health system innovations increasingly being tied to AI, public health outcomes will be increasingly determined by how algorithms, and treatments enabled by these algorithms, are developed. As key actors in the AI development pipeline, developers have important roles to play in developing treatments which are not only effective but are designed in an equitable manner to minimise the potential for harm. Developers are not the only actors responsible for the ethical development and use of AI-enabled healthcare treatments. Scientists, healthcare practitioners, companies, and regulators, among others, all play important parts in the responsible use of AI. However, developers do have key roles due to their capacity to shape the direction of AI-enabled healthcare and by directing their efforts in ways that reflect the values of the wider community. As AI becomes ubiquitous, and is deployed in the context of more personalised forms of medicine, it is vital that developers of these kinds of AI-enabled treatments are aware of the unique challenges that these technologies pose and mitigate the biases that come with their use whenever possible.

Many questions remain about just how feasible it is for developers to implement many of the bias mitigation strategies which are commonly cited in the literature. Actions occurring both upstream and downstream in the development process can have significant impacts on a developer’s ability to provide the basis for non-biased treatments. Developers are often limited by time and resource constraints which may prevent them from undertaking certain bias mitigation activities. They may also lack oversight during the implementation stage. Developers should therefore be encouraged to engage with other actors along the development pipeline, such as healthcare practitioners, who can aid them in incorporating the values and needs of the community into their work. Ambiguity also remains about the appropriate course of action that developers should take in the context of more novel methods such as the use of synthetic data to supplement gaps in existing datasets.

While we have outlined some of the challenges which developers face in mitigating bias in the development of AI-enabled treatments, more research is needed to test the utility and practicality of bias mitigation strategies in real-world settings. More research is required on uses of AI within public health domains where treatments will become increasingly personalised and where patients will have the opportunity to be more actively engaged in their own healthcare. Greater clarity is also needed around the extent of developers’ responsibility and how our notions of responsibility should be shaped by the practical limitations associated with bias mitigation. Are developers still responsible for biases that emerge during model deployment, even if they no longer have oversight over the implementation stage? Should developers be responsible if an algorithm performs poorly on certain subpopulations if data on such populations are absent in the first place? These types of questions must be answered for developers to appropriately direct their efforts to foster reduction of bias in ways that align with their expected responsibilities.

Questions around responsibility are further complicated by the lack of clarity around how developers themselves should be defined. Developers are generally viewed as a homogeneous group of AI experts who are responsible for handling the technical aspects of algorithm design. Yet there are many instances where a scientist may use existing algorithms to develop a given treatment without themselves being experts in algorithm design or having engaged the services of someone traditionally considered to be an AI developer. Scientists may also be hesitant to label themselves as developers, even though their work may involve the customisation of existing algorithms to suit their needs. More research is thus needed to unpack the different types of AI developers and the impact that different development pipelines have in terms of attributing individual responsibilities. Having greater understanding of the wide range of ways in which AI is being used in the public health domain will ultimately enable us to more effectively target bias mitigation strategies to mitigate effects for specific types of AI use. Taking into account the practical challenges that developers may face will enable us to develop methods to overcome these challenges and better assign responsibility for bias mitigation in ways that more accurately reflect the contexts within which developers work.

## Data Availability

The original contributions presented in the study are included in the article/supplementary material, further inquiries can be directed to the corresponding author.

## References

[1] Uddin R Koo I. Real-time remote patient monitoring: a review of biosensors integrated with multi-hop IoT systems via cloud connectivity. Appl Sci. (2024) 14:1876. doi: 10.3390/app14051876

[2] Bhatia D Paul S Acharjee T Ramachairy SS. Biosensors and their widespread impact on human health. Sens Int. (2024) 5:100257. doi: 10.1016/j.sintl.2023.100257, 41406507

[3] Demir G Yegin Z. Artificial intelligence: its potential in personalized public health strategies and genetic data analysis: a narrative review. Pers Med. (2025) 22:171–9. doi: 10.1080/17410541.2025.2494501, 40259534

[4] Dinc R Ardic N. The next frontiers in preventive and personalized healthcare: artificial intelligent-powered solutions. J Prev Med Public Health. (2025) 58:441–52. doi: 10.3961/jpmph.25.080, 40534362 PMC12530966

[5] Clarke L. Synthetic biology, engineering biology, market expectation. Eng Biol. (2020) 4:33–36. doi: 10.1049/enb.2020.0021, 36968158 PMC9996698

[6] Department for Science, Innovation and Technology. National Vision for engineering biology [internet]. UK Government; (2023). Available online at: https://www.gov.uk/government/publications/national-vision-for-engineering-biology/national-vision-for-engineering-biology (Accessed October 24, 2025).

[7] Du Y Jamasb AR Guo J Fu T Harris C Wang Y . Machine learning-aided generative molecular design. Nat Mach Intell. (2024) 6:589–604. doi: 10.1038/s42256-024-00843-5, 41397995

[8] Jain KK. Synthetic biology and personalized medicine. Med Princ Pract. (2013) 22:209–19. doi: 10.1159/000341794, 22907209 PMC5586729

[9] Chen Y Clayton EW Novak LL Anders S Malin B. Human-centered design to address biases in artificial intelligence. J Med Internet Res. (2023) 25:e43251. doi: 10.2196/43251, 36961506 PMC10132017

[10] Flores L Kim S Young SD. Addressing Bias in artificial intelligence for public health surveillance. J Med Ethics. (2024) 50:190–4. doi: 10.1136/jme-2022-108875, 37130756

[11] Chassang G Béranger J Rial-Sebbag E. The emergence of AI in public health is calling for operational ethics to Foster responsible uses. Int J Environ Res Public Health. (2025) 22:568. doi: 10.3390/ijerph22040568, 40283793 PMC12027014

[12] De Silva D Alahakoon D. An artificial intelligence life cycle: from conception to production. Patterns. (2022) 3:100489. doi: 10.1016/j.patter.2022.100489, 35755876 PMC9214328

[13] Harms RJ Ankeny RA Carter L Mankad A Scully JL. Problems with a one-size-fits-all approach: a systematic literature review on solutions to AI bias in engineering biology. SocArXiv [Preprint] (2025). Available online at: https://osf.io/preprints/socarxiv/72bg9_v1 (Accessed December 10, 2025).

[14] Clark CR Wilkins CH Rodriguez JA Preininger AM Harris J DesAutels S . Health care equity in the use of advanced analytics and artificial intelligence technologies in primary care. J Gen Inter Med. (2021) 36:3188–93. doi: 10.1007/s11606-021-06846-x, 34027610 PMC8481410

[15] Johnson AE Brewer LC Echols MR Mazimba S Shah RU Breathett K. Utilizing artificial intelligence to enhance health equity among patients with heart failure. Heart Fail Clin. (2022) 18:259–73. doi: 10.1016/j.hfc.2021.11.001, 35341539 PMC8988237

[16] Panch T Mattie H Atun R. Artificial intelligence and algorithmic: implications for health systems. J Glob Health. (2019) 9:020318. doi: 10.7189/jogh.09.020318, 31788229 PMC6875681

[17] Silano JA. Towards abundant intelligences: considerations for indigenous perspectives in adopting artificial intelligence technology. Healthc Manag Forum. (2024) 37:329–33. doi: 10.1177/08404704241257144, 38830634 PMC11348629

[18] Ellen JG Matos J Viola M Gallifant J Quion J Celi LA . Participant flow diagrams for health equity in AI. J Biomed Inform. (2024) 152:104631. doi: 10.1016/j.jbi.2024.104631 38548006

[19] Straw I Callison-Burch C. Artificial intelligence in mental health and the biases of language based models. PLoS One. (2020) 15:12. doi: 10.1371/journal.pone.0240376, 33332380 PMC7745984

[20] Faghani S Khosravi B Zhang K Moassefi M Jagtap JM Nugen F . Mitigating bias in radiology machine learning: 3. Performance metrics. Radiol Artif Intell. (2022) 4:5. doi: 10.1148/ryai.220061, 36204539 PMC9530766

[21] Kostick-Quenet KM Cohen IG Gerke S Lo B Antaki J Movahedi F . Mitigating racial in machine learning. J Law Med Eth. (2022) 50:92–100. doi: 10.1017/jme.2022.13, 35243993 PMC12140104

[22] Liu M Ning Y Teixayavong S Mertens M Xu J Ting DSW . A translational perspective towards clinical AI fairness. npj Digit Med. (2023) 6:172. doi: 10.1038/s41746-023-00918-4, 37709945 PMC10502051

[23] Pillai M Griffin AC Kronk CA McCall T. Toward community-based natural language processing (CBNLP) with communities. J Med Internet Res. (2023) 25:e48498. doi: 10.2196/48498, 37540551 PMC10439463

[24] Rouzrokh P Khosravi B Faghani S Moassefi M Vera Garcia DV Singh Y . Mitigating bias in radiology machine learning: 1. Data handling. Radiol Artif Intell. (2022) 4:5. doi: 10.1148/ryai.210290, 36204544 PMC9533091

[25] Hane CA Wasserman M. Designing equitable health care outreach programs from machine learning patient risk scores. Med Care Res Rev. (2023) 80:216–27. doi: 10.1177/10775587221098831, 35685000

[26] de Biase A Sourlos N van Ooijen PMA. Standardization of artificial intelligence development in radiotherapy. Semin Radiat Oncol. (2022) 32:415–20. doi: 10.1016/j.semradonc.2022.06.010, 36202443

[27] Felzmann H Fosch-Villaronga E Lutz C Tamò-Larrieux A. Towards transparency by design for artificial intelligence. Sci Eng Ethics. (2020) 26:3333–61. doi: 10.1007/s11948-020-00276-4, 33196975 PMC7755865

[28] Corpas M Pius M Poburennaya M Guio H Dwek M Nagaraj S . Bridging genomics’ greatest challenge: the diversity gap. Cell Genom. (2025) 5:1. doi: 10.1016/j.xgen.2024.100724, 39694036 PMC11770215

[29] Getzen E Ungar L Mowery D Jiang X Long Q. Mining for equitable health: assessing the impact of missing data in electronic health records. J Biomed Inform. (2023) 139:104269. doi: 10.1016/j.jbi.2022.104269, 36621750 PMC10391553

[30] Jukarainen S Kiiskinen T Kuitunen S Havulinna AS Karjalainen J Cordioli M . Genetic risk factors have a substantial impact on healthy life years. Nat Med. (2022) 28:1893–901. doi: 10.1038/s41591-022-01957-2, 36097220 PMC9499866

[31] Mauvais-Jarvis F Bairey Merz N Barnes PJ Brinton RD Carrero J-J DeMeo DL . Sex and gender: modifiers of health, disease, and medicine. Lancet. (2020) 396:565–82. doi: 10.1016/S0140-6736(20)31561-0, 32828189 PMC7440877

[32] Chen W Yang K Yu Z Shi Y Chen CLP. A survey on imbalanced learning: latest research, applications and future directions. Artif Intell Rev. (2024) 57:137. doi: 10.1007/s10462-024-10759-6

[33] Vandersluis R Savulescu J. The selective deployment of AI in healthcare: an ethical algorithm for algorithms. Bioethics. (2024) 38:391–400. doi: 10.1111/bioe.13281, 38554069 PMC7616300

[34] Whitney CD Norman J. Real risks of fake data: synthetic data, diversity-washing and consent circumvention. In: FAccT 24: Proceedings of the 2024 ACM conference on fairness, accountability, and transparency Jun 3–6; (2024); Rio de Janeiro, 1733–44. Available online at: https://facctconference.org/static/papers24/facct24-117.pdf (Accessed October 24, 2025).

[35] Orr W Crawford K. Building better datasets: seven recommendations for responsible design from dataset creators. J Data-centric Mach Learn Res. (2024) 1:1. doi: 10.48550/arXiv.2409.00252

[36] Casaletto J Bernier A McDougall R Cline MS. Federated analysis for privacy-preserving data sharing: a technical and legal primer. Annu Rev Genomics Hum Genet. (2023) 24:347–68. doi: 10.1146/annurev-genom-110122-084756, 37253596 PMC10846631

[37] Bhanbhro J Nisticò S Palopoli L. Issues in federated learning: some experiments and preliminary results. Sci Rep. (2024) 14:29881. doi: 10.1038/s41598-024-81732-0, 39623121 PMC11612434

[38] Fiske A Blacker S Geneviève LD Willem T Fritzsche M-C Buyx A . Weighing the benefits and risks of collecting race and ethnicity data in clinical settings for medical artificial intelligence. Lancet. (2025) 7:e286–94. doi: 10.1016/j.landig.2025.01.003, 40148011

[39] Visweswaran S Sadhu EM Morris MM Vis AR Samayamuthu MJ. Online database of clinical algorithms with race and ethnicity. Sci Rep. (2025) 15:10913. doi: 10.1038/s41598-025-94152-5, 40157976 PMC11954862

[40] Bazzano AN Mantsios A Mattei N Kosorok MR Culotta A. AI can be a powerful social innovation for public health if community engagement is at the core. J Med Internet Res. (2025) 27:e68198. doi: 10.2196/68198, 39841529 PMC11799803

[41] Timmons AC Duong JB Simo Fiallo N Lee T Vo HPQ Ahle MW . A call to action on assessing and mitigating in artificial intelligence applications for mental health. Perspect Psychol Sci. (2023) 18:1062–96. doi: 10.1177/17456916221134490, 36490369 PMC10250563

[42] Bavli I Galea S. Key considerations in the adoption of artificial intelligence in public health. PLOS Digit Health. (2024) 3:7. doi: 10.1371/journal.pdig.0000540, 38950051 PMC11216572

[43] Pratte MM Audette-Chapdelaine S Auger A-M Wilhelmy C Brodeur M. Researchers’ experiences with patient engagement in health research: a scoping review and thematic synthesis. Res Involv Engagem. (2023) 9:22. doi: 10.1186/s40900-023-00431-8, 37038164 PMC10088213

[44] Nadarzynski T Knights N Husbands D Graham CA Llewellyn CD Buchanan T . Achieving health equity through conversational AI: a roadmap for design and implementation of inclusive in healthcare. PLOS Digit Health. (2024) 3:5. doi: 10.1371/journal.pdig.0000492, 38696359 PMC11065243

[45] Parischa S. AI ethics in smart healthcare. IEEE Consum Electron Mag. (2023) 12:12–20. doi: 10.1109/MCE.2022.3220001

[46] Hasanzadeh F Josephson CB Waters G Adedinsewo D Azizi Z White JA. Bias recognition and mitigation strategies in artificial intelligence healthcare applications. npj Digit Med. (2025) 8:154. doi: 10.1038/s41746-025-01503-7, 40069303 PMC11897215

[47] Riva M Parigi TL Ungaro F Massimino L. Hugging face’s impact on medical applications of artificial intelligence. Comput Struct Biotechnol Rep. (2024) 1:100003. doi: 10.1016/j.csbr.2024.100003

[48] Al-Kharusi Y Khan A Rizwan M Bait-Suwailam MM. Open-source artificial intelligence privacy and security: a review. Computers. (2024) 13:311. doi: 10.3390/computers13120311

[49] Page MJ McKenzie JE Bossuyt PM Boutron I Hoffmann TC Mulrow CD . The PRISMA 2020 statement: an updated guideline for reporting systematic reviews. BMJ. (2021) 372:71. doi: 10.1136/bmj.n71, 33782057 PMC8005924

